# Supplementary material for: Patterns of Intron Gain and Loss in Fungi
Source: PLoS Biol. 2004 Nov 30;2(12):e422. doi: 10.1371/journal.pbio.0020422 (PMC532390; doi:10.1371/journal.pbio.0020422)
Supplement: Table S1 — Also available at http://genes.mit.edu/NielsenEtAl/. (4.3 MB ZIP). [file pbio.0020422.st001.zip › NielsenEtAl/html/970.html]

AN3034.1.NCU06086.1.MG00843.1.FG07041.1


```
 CLUSTAL W (1.82) Multiple Sequence Alignments - Introns Inserted


Sequence 1: NCU06086.1	274 aa
Sequence 2: FG07041.1	290 aa
Sequence 3: MG00843.1	293 aa
Sequence 4: AN3034.1	303 aa
Alignment Length: 313 aa
Number Identitical Residues: 75 aa
Alignment Score (without introns) 4330


MG00843.1 	MMSLRTFAR-SAPRALSRVSSAAAYRTTGSPFL--KTSSFADSSLKAASR--PAAFSTSP
NCU06086.1	MMSLRAVARTTAPRALRAVSASPITLGR-------ATVRATSNVLRTQLP--QKAFSTTV
FG07041.1 	MFSARNVAR-SAPRVVSRLSGAALRQTA-------RPSAFIKASSARRPA--QAAFSTTV
AN3034.1  	MLSLRTLTR-TVPRTFSRSIATSSRSAILRPISNLPKTSFIQPSLKQAIRPSQAAFSTCK
          	*:* * .:* :.**..    .:.       .  .       .        .:  ****  

MG00843.1 	LRRAQNAV~DEELANKLDQELSFEAE--VKKSEPEPAIIKDFLENGPWE~LKDVSGQEKV
NCU06086.1	FRAAATEI~DSELSEKLSTEIGYEQD--IASNEPVPATVKDFLENGPFE~LVDTPGKEDV
FG07041.1 	FRKAA-EN~DGELLAKLESEIKIEED--MKADEQDPASIKDFLNNSAFE~LIDTPGQEVV
AN3034.1  	AFRQA-EG1DAELAAKLEDELKHEKSSGLEDLESSVQNIQYVLQNNSWE0VKDVPGEQEV
          	         * **  **. *:  * .:.:   *     :: .*:*..:* : *..*:: *

MG00843.1 	VLTRSFDNEK2ITLTFSIADLATEYHDQEADTYDSDPALADESADSKGRSNEIEN-----
NCU06086.1	VLKRTFGNEQ2ITISFSIADLQ----NYEPGMFDEDTALGDE----EGAQN---------
FG07041.1 	KLVRNFGEEK2ITISFSIADIT----SYDP--YAEENGLEEEGFEDEGAQGSQRNARANE
AN3034.1  	VLTKKFNDEE2IRLTFTVADLQNLSEHEDLDALDELDYQGHQPVNQGGAGGFAQHPE---
          	 * :.*.:*: * ::*::**: .     : .   .     .:  .. *  .    .    

MG00843.1 	-----AEEEYDEQDAAPP--CRLNVVIEKPNSGKGALNIEATLQDDQLAVENVYYFDSAE
NCU06086.1	------EEDMGEDGGAP---VRLNIVIEKPSK--GALNIDAVAQDGSIVVDNMFYYHDAK
FG07041.1 	ELEEELDEEAEEENAAP---INLSIVVEKPGKASGALNIDATAQDGHIVVENLFYYDDAK
AN3034.1  	--DSVSPADRGEQDLEPSFPARVNVTIEKSGK--GALLIQTVAQDGLFQVEEVSYFSKPD
          	  ..    :  *:.  *. . .:.:.:**...  *** *::. **. : *::: *: ...

MG00843.1 	LAKEESAETAHKASNIYPGPPFGTLDEELQVLMERYLEERGVTADLAVFVPQYMDVKEQR
NCU06086.1	LAHGESAETQHAAQAVYPGPPFGTLDEDLQVLMERYLEDRGINQALALFVPDYLDMKEQK
FG07041.1 	VAKVESPEAAQKRADVYPGPPFGSLDDDLQVLMERFLEERGITQALAIFVPDYVDVKEQR
AN3034.1  	LAHAATAEKDWARQSLYAGPPFENLDEDLQTYLERYLEERGINAELANMIPDYIQVKEQK
          	:*:  :.*       :*.**** .**::**. :**:**:**:.  ** ::*:*:::***:

MG00843.1 	EYTNWLKN~VKDFVKA-
NCU06086.1	EYLRWLNN~LKGFVDAQ
FG07041.1 	EYTRWLNN~VKAFIDA-
AN3034.1  	EYVRWLEN1VRNFVAA-
          	** .**:* :: *: *
```
